# Vertical clinging and leaping induced evolutionary rate shifts in postcranial evolution of tamarins and marmosets (Primates, Callitrichidae)

**DOI:** 10.1186/s12862-021-01848-z

**Published:** 2021-06-25

**Authors:** Léo Botton-Divet, John A. Nyakatura

**Affiliations:** AG Vergleichende Zoologie, Institut Für Biologie, Humboldt-Universität Zu Berlin, Philippstr. 13, 10115 Berlin, Germany

**Keywords:** Platyrrhini, Femur, Humerus, Leaping, Geometric morphometrics

## Abstract

**Background:**

Callitrichids comprise a diverse group of platyrrhine monkeys that are present across South and Central America. Their secondarily evolved small size and pointed claws allow them to cling to vertical trunks of a large diameter. Within callitrichids, lineages with a high affinity for vertical supports often engage in trunk-to-trunk leaping. This vertical clinging and leaping (VCL) differs from horizontal leaping (HL) in terms of the functional demands imposed on the musculoskeletal system, all the more so as HL often occurs on small compliant terminal branches. We used quantified shape descriptors (3D geometric morphometrics) and phylogenetically-informed analyses to investigate the evolution of the shape and size of the humerus and femur, and how this variation reflects locomotor behavior within Callitrichidae.

**Results:**

The humerus of VCL-associated species has a narrower trochlea compared with HL species. It is hypothesized that this contributes to greater elbow mobility. The wider trochlea in HL species appears to correspondingly provide greater stability to the elbow joint. The femur in VCL species has a smaller head and laterally-oriented distal condyles, possibly to reduce stresses during clinging. Similarly, the expanded lesser trochanters visible in VCL species provide a greater lever for the leg retractors and are thus also interpreted as an adaptation to clinging. Evolutionary rate shifts to faster shape and size changes of humerus and femur occurred in the *Leontocebus* clade when a shift to slower rates occurred in the *Saguinus* clade.

**Conclusions:**

Based on the study of evolutionary rate shifts, the transition to VCL behavior within callitrichids (specifically the *Leontocebus* clade) appears to have been an opportunity for radiation, rather than a specialization that imposed constraints on morphological diversity. The study of the evolution of callitrichids suffers from a lack of comparative analyses of limb mechanics during trunk-to-trunk leaping, and future work in this direction would be of great interest.

**Supplementary Information:**

The online version contains supplementary material available at 10.1186/s12862-021-01848-z.

## Background

Facilitated by the collections in natural history museums from around the globe, the comparative analysis of mammalian limb long bones is an important means of gaining insight into phenotypic evolutionary disparity and diversification (e.g., [1–11]). Bone shape reflects adaptations to specific functional demands related to taxon ecology, as well as being constrained by body size (allometric changes) and phylogenetic history [7, 12–14]. The study of functional shape in respect to organismal evolution has been facilitated by the advent of three-dimensional (3D) imaging techniques and improvements and availability of software for multivariate statistics, including being able to account for the phylogenetic relatedness of analyzed specimens [13, 15–18].

Although the primate postcranial skeleton is relatively conservative in terms of the number, and the organization, of bones (e.g. in comparison to bats, cetaceans, or perissodactyls), primates have nevertheless evolved a wide variety of locomotor behaviors [19–22]. The locomotor repertoire can also be relatively diverse even within individual primate species [23]. Tamarins and marmosets (Callitrichidae; Primates), a species-rich clade of New World primates, display striking differences in locomotor and feeding ecology despite a lack of notable morphological differences. Comparative analysis of 3D limb long bone shape has the potential to detect subtle phenotypic differences between species and is employed here to gain insight into the ecologically significant disparity and evolution within Callitrichidae.

### Callitrichidae

The Callitrichidae, (also sometimes classified as sub-family Callitrichinae [24–26]) constitute a group of over 60 species and subspecies [27] living across South and Central America [28]. All species are of a relatively small size, with head and body lengths ranging from just 130 to 370 mm [28] and body masses of 105 to 700 g [24]. Callitrichids also possess secondarily pointed claws (tegulae) instead of flat nails on all digits except the hallux, and exhibit lateral sequence gaits instead of the diagonal sequences usually used by quadrupedal primates. Due to these combined characteristics, members of the Callitrichidae have been proposed as viable models for certain hypothetical stages of early primate evolution (see [29], for a review). Diurnality and arboreality are also collective traits across all callitrichid species [28].

### Locomotor behavior of Callitrichidae

**Fig. 1:**
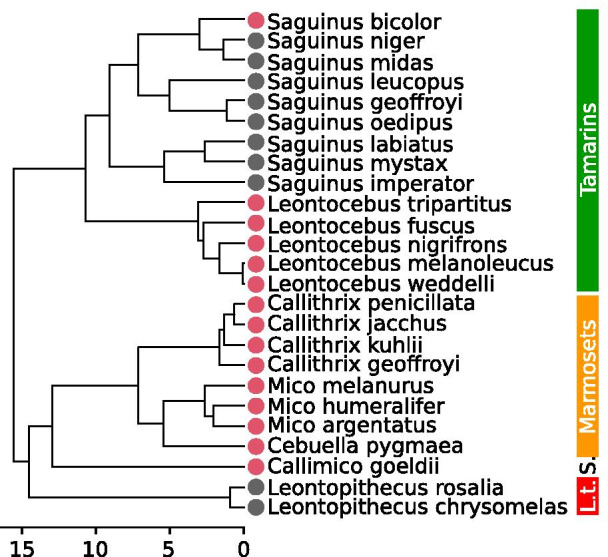
Phylogeny of the callitrichid species considered in the present study. Based on Aristide et al. ([73]; see Material and methods for details). Scale in millions of years (My). Red dots, vertical clingers and leapers (VCL); black dots, horizontal leapers (HL). Common name of groups are provided on the right; L.t.: lion tamarins; S.: spring tamarin

In contrast to the shared ecological traits described above, locomotor behavior differs greatly between callitrichid taxa [23, 30, 31]. Some species use vertical clinging and leaping (VCL) and make extensive use of vertical supports, employing trunk-to-trunk leaping to travel, forage or escape predators. VCL species, such as the spring tamarin *Callimico goeldii*, are often found in forest layers close to the ground [32]. Other callitrichids preferentially use horizontal leaping (HL), and leap from and usually land on thin, flexible, terminal branches in the canopy, such as lion tamarins of the genus *Leontopithecus* [33]. Across Callitrichidae a continuous spectrum exists, ranging from either VCL or HL specialists to various intermediate degrees of tropism (Table 1 and references therein). To a certain extent this locomotor diversity is linked to diversity in feeding behavior [24, 34, 35]. VCL is often used to facilitate various types of foraging on large tree trunks, including gummivory, and foraging for fungi, and insects in bark, whereas terminal branch locomotion and leaping are associated with fruit and nectar exploitation, as well as feeding on insects in the canopy, or small vertebrates [24, 36–38]. VCL is common in the subclade formed by *Callimico*, *Cebuella*, *Mico*, and *Callithrix*, and is also exhibited by some tamarin species [24, 39–41]. A predominant use of VCL is therefore not restricted to a single lineage, so it therefore is possible to study two independent adaptive specializations toward VCL (Fig. 1). Moreover, preference in the orientation and diameter of the support used while leaping appears to be decoupled from the environment, as illustrated by the behavior of mixed-species groups comprised of species with different specializations. The use of the substrate is therefore apparently a matter of species’ preference and differential advantage, rather than substrate availability [39, 42–45].

**Table 1:** Informations on the species ecology and morphology gathered from literature

Species	Locomotion				Food						IMI		Body mass			Cat	
	VCL% of leaps	VCL% of loco	Leaps% of loco	Ref	Fruit flower (%)	Exudates (%)	Animal prey (%)	Fungi (%)	Other (%)	Ref	IMI	Ref	M	F	Ref		Compl. Ref
*Callimico goeldii*	55.1		45	[39]	30	0	27	39	4	[46]	69	[47]	499	468	[48]	VCL	
	63			[49]	14	20	13	52	1	[50]							
					29	1	34	29	7	[34]							
*Callithrix geoffroyi*					15	68	15	0	2	[51]			359		[48]	VCL	
*Callithrix jacchus*					30	40	30	0	0	[52]	76	[47]	317	324	[48]	VCL	
					22	59	19	0	0	[53]							
*Callithrix kuhlii*					27	35	38	0	0	[54]			375		[48]	VCL	
					28	14	58	0	0	[55]							
*Callithrix penicillata*					0	71	29	0	0	[56]	76	[47]	344	307	[48]	VCL	
*Cebuella pygmaea*	37		24.4	[31]	0	77	23	0	0	[57]	83	[47]	110	122	[48]	VCL	[110]
*Leontopithecus chrysomelas*					91	1	8	0	0	[58]			620	535	[48]	HL	[24, 136]
*Leontopithecus rosalia*	8.9	2.8		[33]	84	2	14	0	0	[59]	89	[47]	620	598	[48]	HL	[24]
					82	1	16	0	1	[59]							[110]
*Leontocebus fuscicollis*					42	11	48	0	0	[60]	79	[47]	343	358	[48]	VCL	[110]
					56	12	26	0	6	[34]							
*Leontocebus fuscus*																VCL	[39]
*Leontocebus melanoleucus*																VCL	
*Leontocebus nigrifrons*	54.5	24		[61]	27	9	62	0	2	[62]			413	412	[63]	VCL	
*Leontocebus tripartitus*	17.5	5.8	33.7	[31]	49	11	37	0	3	[60]	80	[47]				VCL	
*Leontocebus weddelli*	20	6.7	~ 38.5	[39]	71	16	8	0	5	[64]	81	[65]				VCL	[43]
	67.5			[46]													
*Mico argentatus*					36	59	5	0	0	[66]	76	[47]	330	360	[48]	VCL	[63]
*Mico humeralifer*					83	17	0	0	0	[38]	76	[65]	475	472	[48]	VCL	[135]
*Mico melanurus*																VCL	
*Saguinus bicolor*					40	1	59	0	0	[67]			428	430	[48]	VCL	[109]
*Saguinus geoffroyi*		4		[68]	38	14	40	0	7	[36]	76	[47]	482	430	[48]	HL	
	3.8			[40]													
*Saguinus imperator*	11.4			[49]	33	0	67	0	0	[69]	75	[47]	474	475	[48]	HL	[110]
*Saguinus labiatus*	8.4		38	[39]	73	8	11	0	8	[34]	73	[47]	490	529	[48]	HL	
	11.9			[49]													
*Saguinus leucopus*					83	0	15	0	2	[70]	74	[47]	494	490	[48]	HL	
*Saguinus midas*			26	[41]	63	0	31	0	6	[47]	77	[47]	515	575	[48]	HL	
	7.3	1.9		[33]													
*Saguinus mystax*	8.8	2.7		[40]	50	10	40	0	0	[60]	74	[47]	510	539	[48]	HL	[30, 113]
*Saguinus niger*					88	3	9	0	0	[71]			490		[71]	HL	[108]
*Saguinus oedipus*					64	24	10	2	0	[72]	74	[47]	418	404	[48]	HL	[36]

VCL% of leaps: percentage of leaps that are trunk to trunk leaps; VCL% of loco.: trunk-to-trunk leaping as percentage of the locomotion; Leaps % of loco.: leaping as percentage of the locomotion; Ref.: reference from which the figures are extracted; IMI: intermembral index; M: males; F: females; Compl. Ref.: complementary references. *Leontocebus fuscicollis,* which is not included in the study, appear for comparison with other species of the genus

### Morphological correlates to locomotor behaviour

Specific morphological adaptation to VCL has been extensively studied in Strepsirrhini [74–76], and callitrichid VCL kinematics, mechanics, and ecology have also been studied [24, 33, 75, 77–79]. Still, morphological correlates of adaptations to this type of locomotion in Callitrichidae have received limited attention, and previous work was focused on functional interpretations of fossils or poorly known species [80, 81], or on hand and feet morphology [42, 68]. The intermembral index (IMI) obtained from the ratio of forelimb length (humerus + radius): hindlimb length (femur + tibia) [23] has been suggested as a marker of specialization to VCL [39, 76, 82], and it has been demonstrated that a proportionally longer hind-limb (i.e. a low IMI) is linked to greater take-off speed [78]. Garber and Leigh [39] showed that *Callimico goeldii* has extremely elongated hind-limbs, a characteristic also observed in Strepsirrhini. The authors also described a long patellar groove in *Leontocebus fuscicollis* and interpreted it as an adaptation to greater knee excursion during vertical clinging and leaping. Nevertheless, Garber and Leigh [39] measured a high IMI in *Leontocebus fuscicollis*, mostly because of the elongated forearm, which increases when the hand is included. They discussed it also as an adaptation to trunk-to-trunk leaping, the relative long forelimb providing a greater distance when flexing the limb to dissipate the landing impact, and the mass distribution farther from the bodyprovides an advantage during in-air rotation. Similarly, Falsetti and Cole [82] suggested that the greater IMI in *Leontocebus fuscicollis* compared to *Callithrix jacchus* and *Saguinus oedipus* might increase agility on trunks, with the greater forelimb span facilitating clinging and deceleration during landing. But, as detailed above, a lower IMI has also been suggested to be beneficial for leaping (a relatively longer hindlimb allowing for a longer distance during propulsion) indicating that the intermembral index is limited as an explanatory variable for differences in locomotor evolution among Callitrichidae. There may be different evolutionary pathways behind the independently acquired VCL behavior in *Leontocebus* and in the *Callithrix*/*Callimico*/*Mico*/*Cebuella* clade, potentially reflected in subtle differences of limb long bone shape between both taxa.

Bicca-Marques [42] showed that *Leontopithecus* have long and slender hands, and described a similar pattern in *Leontocebus fuscicollis*, *Leontocebus tripartitus*, and *Leontocebus nigricollis*. However these characteristics are discussed in respect to feeding strategies, not locomotion. Smith and Smith [68] showed a link between foot length, tegulae curvature, and the use of vertical supports. They also demonstrated the increasing number of plantar and palmar ridges in relation to the degree of exudativory.

### Differences in functional demands posed by VCL and HL

VCL differs from HL in the mechanical demands faced by the animal, especially in respect to the limb long bones. Generation of leaping forces during trunk-to-trunk leaping mostly involves the hindlimbs [77, 78]. The flight phase during VCL also involves a change in body orientation to face the landing support, which is not necessary during HL. Landing on non-compliant supports implies high compressive forces as all the landing energy is actively dissipated during leg flexion. During HL, the majority of the landing supports are highly compliant, making it necessary to grasp and balance rather than to dissipate impact forces. The evolutionary role of primate proximal limb long bones (i.e., the humerus and femur) in locomotor behavior is considered to be very important [83–87]. All mechanical loads transmitted between the limb and the tree trunk support pass via these bones. The bone morphology can therefore be expected to reflect different functional demands related to locomotor behavior, and thus provide insight into how morphology relates to ecology and may reflect the evolution of the entire family.

### Evolutionary rates

The study of evolutionary rates (both molecular and phenotypic) is key for the understanding of macroevolution, and is the subject of much debate and methodological development [17, 88–93]. The rhythm of evolutionary change is a useful indicator of either the acquisition of a new fitness optimum, or of a strong stabilizing selection acting on the system. The adaptive radiation model [93] predicts a higher evolutionary rate in response to the emergence of a new function, or during the selection process in response to a new or changing environment. In the case of Callitrichidae, the development of VCL locomotion is hypothesized to coincide with an increase in the evolutionary rate of the locomotor structures involved.

### Aims

Morphological differences for femur and humerus between VCL and HL species are investigated. Significant differences in shape are expected (e.g. reinforced hindlimb extensors insertion in VCL), and the size difference between the groups is also considered in order to identify any deviations from isometric shape change with increasing body size (allometry). The differences themselves are described and their functional significance discussed. Finally, as the exploitation of a new ecological niche is expected to provide the opportunity for rapid diversification, changes in the evolutionary rates across Callitrichidae are investigated in order to determine if VCL has been a driver of increased morphological diversification. All of these questions are analyzed while accounting for body size and accounting for the statistical non-independence of taxa due to their phylogenetic relatedness using multivariate statistical and phylogenetic comparative methods.

## Results

### Size

We observed a significant phylogenetic signal for centroid size for both the humerus (K = 0.832, p-value = 1e−04) and the femur (K = 0.680, p-value = 0.0022).

#### Differences between ecological groups

**Fig. 2:**
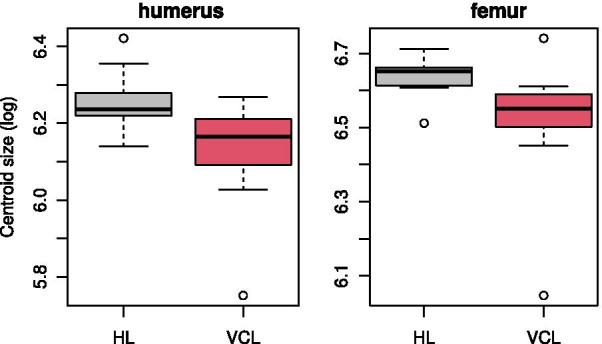
Boxplot of the log-transformed centroid size per ecological category for humerus and femur

For both the humerus and femur, VCL species have a smaller centroid size compared to HL species (t-test on log-transformed centroid size: p-value = 0.0075 and 0.0128 for the humerus and femur, respectively). However there is an overlap in the size distribution of the two groups (Fig. 2) and when phylogeny is taken into account the differences are no longer significant (phylogenetic ANOVA on 1000 simulations for humerus: p-value = 0.251, for femur: p-value = 0.298).

#### Per taxa centroid size difference

**Fig. 3:**
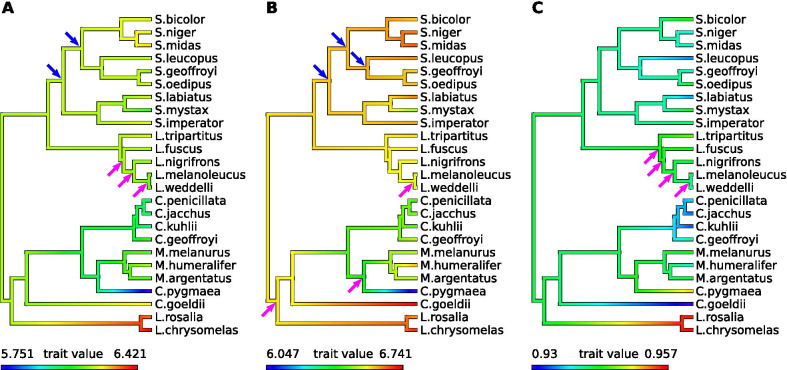
Mapping of the log-transformed centroid size and centroid size ratio. Centroid size of **A** humerus; **B** femur and **C** ratio of humeral centroid size (log) over femoral centroid size (log), over the phylogeny. Arrows stand for significant rate shifts: purple arrows for a rates significantly in the clade than in the rest of the tree, blue for lower rates

The tamarins (*Saguinus* and *Leontopithecus*) are distinct from the other species in our sample in that they have a relatively homogenous centroid size for both humerus and femur (Fig. 3A, B). *Leontopithecus* specimens present large humerus and femur sizes, whereas *Cebuella* present small values, and *Callithrix* species lie between these two groups. *Callimico* display a medium value for the humerus, but a high value for the femur. *Saguinus midas* and *S. niger* have high femoral centroid sizes. In terms of the ratio of the humeral centroid size over the femoral one (Fig. 3C), *Callimico* exhibits a larger hind limb proximal segment compared to the forelimb (ratio = 0.929). To a lesser extent, this is also the case in *Callithrix* species (ratios between 0.933 and 0.940) and *Saguinus leucopus* and *S. labiatus* (both 0.935). *Leontopithecus* specimens present the highest ratios (0.956–0.957), followed by *Cebuella* (0.951). *Leontocebus* (0.940–0.946), *Mico* (0.939–0.944) and all other *Saguinus* (0.935–0.942) display medium values.

#### Evolutionary rates of centroid size

Evolutionary rates for humeral and femoral size are significantly higher for the inner nodes of the *Leontocebus* clade, and all nodes of the clade show significantly higher rates for the size ratio (Fig. 3A–C). The evolutionary rates for the centroid size of the femur are significantly higher for the node that separates *Leontopithecus* from marmosets and *Callimico*, and for the node that separates *Cebuella pygmaea* from *Mico* species. There is a significantly lower evolutionary rate for humeral size and femoral size for the basal node of *Saguinus*, and for the node that separates the *Saguinus midas* and the *Saguinus goeffroyi* clades. Femoral size is also significantly lower at the node separating *Saguinus leucopus* from the *S.geoffroyi/S.oedipus* clade.

### Shape

We did not observe a significant phylogenetic signal for shape for either the humerus or the femur (K_mult_ of 0.080 and 0.087, p-value of 0.67 and 0.64 for the humerus and femur respectively). No significant deviation from isometric shape in respect to centroid size was found in the humerus, however, the p-value is close to significance. There is significant allometry in femoral shape (Table 2). The locomotor categories have a significant impact on both the humeral and femoral shape. The interaction term that accounts for both size and locomotor mode is significant for the humerus, indicating that the allometric slopes in the humerus differ significantly between the two locomotor groups.

**Table 2:** MANCOVA on shape data (PCs axes totalling 95% of total variance) for centroid size, locomotor categories, and interaction of these

	Df	Pillai	Approx F	Num Df	Den Df	P-value
Humerus						
Size (log)	1	0.83612	2.9154	14	8	0.06615
Locomotion	1	0.86635	3.7043	14	8	**0.03431**
Interaction	1	0.89708	4.9808	14	8	**0.01414**
Residuals	21					
Femur						
Size (log)	1	0.91085	2.9445	15	7	**0.02223**
Locomotion	1	0.91897	5.2928	15	7	**0.01661**
Interaction	1	0.79672	1.8290	15	7	0.21353
Residuals	21					

Significant p-values are in bold

#### Humeral shape

**Fig. 4:**
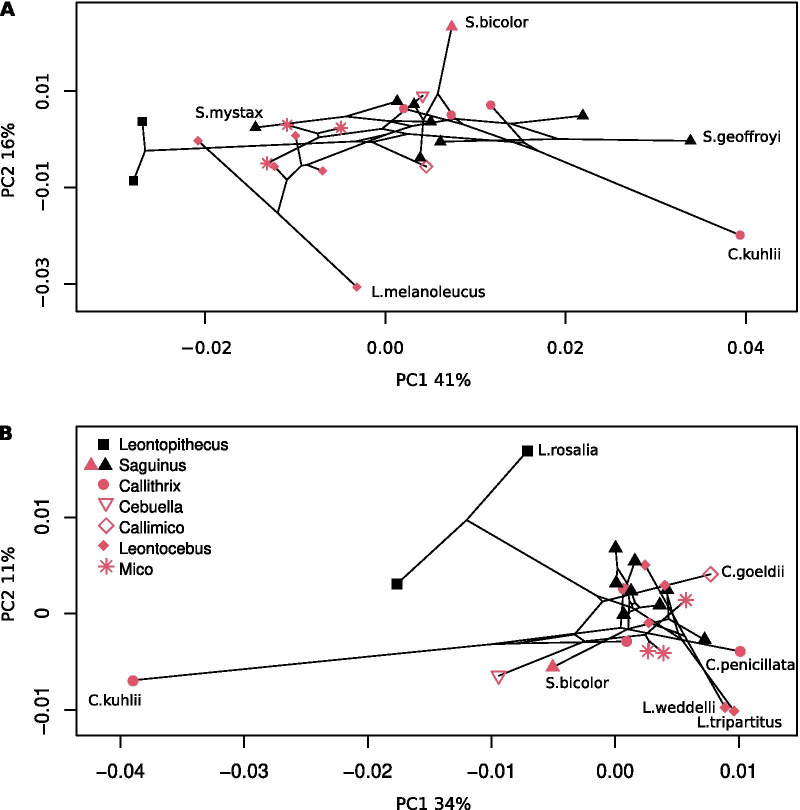
First and second axes of the phylomorphospace. Phylomorphospace computed from: **A** humeral shape; **B** femoral shape. Shape correspond to genus, colour to the locomotor categories: red VCL, black HL

**Fig. 5:**
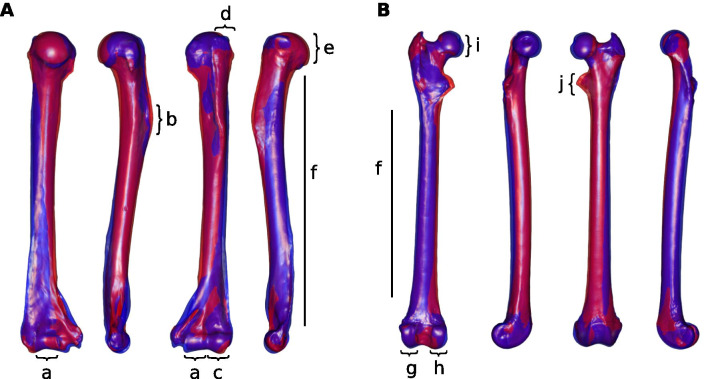
Shape deformation associated with the between-group PCA axis. **A** humerus; **B** femur. For both, views from left to right: caudal, medial, cranial, lateral. a: trochlea, b: deltoid crest, c: capitulum, d: greater tubercle, e: humeral head, f: diaphysis, g: lateral condyle, h: medial condyle, i: femoral head, j: lesser trochanter. In red VCL species, in blue HL species

The first axis (PC1) of the phylomorphospace computed for humeral shape (Fig. 4A) reflects 41% of total variance. *Leontopithecus* plots on the negative end of PC1, whereas *Callithrix kuhllii* and *S. geoffroyi* fall on the extreme positive end. Species of the genera *Leontocebus* and *Mico* plot close to the negative end, as well as *Saguinus mystax*. PC2 (16% of total variance) separates *Saguinus bicolor* on the extreme positive side and *Leontocebus melanoleucus* on the extreme negative. There is no obvious separation between the two locomotor categories along either axis. PC1 on humeral shape separates species with robust bones, a caudally bent diaphysis, and proportionally large epiphyses on the positive side from species with relatively more gracile bones with smaller epiphyses. PC2 on humeral shape separates the species with a diaphysis bent posteriorly around the deltoid tuberosity on the negative side from species with a more straight shaft on the positive side. For the humerus, the between-group PCA separates the locomotor groups with an accuracy of 84%. The p-value of the group differences obtained from permutation testing is 0.3812. The observed differences in shape are a straighter humeral neck in VCL species whereas HL species have a humeral head more caudally curled (Fig. 5A). The medial lip of the trochlea is expanding more medially in HL resulting in a broader cranial area of the trochlea, and the medial supratrochlear area is more expanded. The lateral supracondylar crest has a broader proximal portion in HL species compared to those that use VCL.

#### Femoral shape

The phylomorphospace computed for femoral shape (Fig. 4B) displays along PC1 (34% of total variance) *Callithrix kuhllii* on the extreme negative end from most of the other species. Still, species of the genus *Leontopithecus*, *Cebuella* and *Saguinus bicolor* have lower values on PC1 than all other species, except for *Callithrix kuhllii*. PC2 (11% of total variation) depicts *Leontopithecus rosalia* on the extreme positive end, and *Leontocebus tripartitus* and *Leontocebus weddelli* on the extreme negative end. PC2 appears to tentatively separate the two locomotor categories, with VCL species trending towards lower values, whereas HL species trend towards higher values along this PC axis.

PC1 of femoral shape separates species with proportionally larger epiphyses on the negative end from species with relatively smaller ones on the positive end. PC2 of femoral shape separates species with a proportionally larger femoral head and a smaller lesser trochanter on the positive end of the axis. Please refer to Additional file 1 (Fig. S3) for graphic representation and a more detailed description.

The between-group PCA on the femur (Fig. 5B) separates the groups with an accuracy of 88%, but the p-value of the between-group difference remains non-significant (0.0977). The VCL group differs in shape from the HL by its proportionally smaller femoral head, larger lesser trochanter, and narrower distal part of the medial condyle, with the plane tangent to the most distal point of two condyles tilted toward the medial side.

#### Evolutionary rates of shape changes

**Fig. 6:**
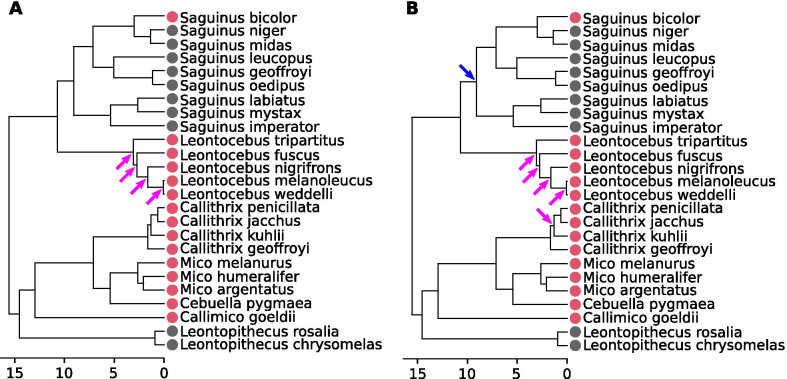
Shifts of evolutionary rates computed on shapes following the method of Castiglione et al. [94]. **A** humerus; **B** femur. Purple arrows depict significantly higher rates, blue arrows depict significantly lower rates. Scale in millions of years (My)

When testing for shifts in evolutionary rates (Fig. 6) we observe significantly higher rates for the evolution of the shape of both femur and humerus in the *Leontocebus* clade. The node separating *Callithrix kuhllii* from *Callithrix penicillata* and *Callithrix jacchus* has a significantly higher evolutionary rate for femoral shape change. Both the crownward and stemward nodes relative to the aforementioned node present high evolutionary rates but are not significant (Additional file 1: Table S4). There is a significantly lower evolutionary rate for femoral shape change for the basal node of *Saguinus*. Values for the other nodes are reported in Additional file 1 (Additional file 1: Table S4 and Fig. S4).

## Discussion

### Evolution of size in callitrichid proximal limb long bones

To gain insight into the evolution of callitrichid primates and specifically to investigate whether differences in locomotor ecology are reflected in proximal limb bone morphology, we compared size and shape within a comparative sample of humeri and femora. Within this clade of primates, VCL has independently evolved twice, under the hypothesis of HL being the ancestral state (see Garber [24] for a synthesis).

#### Size

We observed that VCL species present on average a smaller centroid size for both the humerus and femur compared to HL species. It was noticed in previous work [24] that among callitrichids, VCL species are generally smaller in size than their HL counterparts. Still, as noticed by Garber [24], there is an overlap in the size range of the two ecological categories. In our dataset, the highly specialized VCL *Callimico goeldii* has the largest centroid size for the femur and the fourth largest centroid size for the humerus, behind three HL species. The relatively large body mass of this species was previously noted (male mean 499 gr, female mean 468 gr; [48]) compared to other VCL species like *Callithrix jacchus* (male mean 317 gr, female mean 324 gr; [48]).

The evolution of centroid size is highly influenced by phylogenetic inertia. When mapped across the phylogeny (Fig. 3A), humeral centroid size shows limited variation, except among the *Leontopithecus*, *Callimico*, and *Cebuella* clades. The variations of femoral size are greater across the phylogeny, and accordingly the ratio of humeral to femoral centroid size also varies over the phylogeny.

#### Size ratio

Intermembral Index (IMI) is often seen as a better predictor for locomotion in primates than overall size [74, 95, 96]. As a general trend among primates, highly specialized VCL primates tend to present a low IMI compared to species with quadrupedal habits, the brachiating species scoring higher [23, 97]. Additionally, IMI tends to scale positively with body size, thus the combined effect of body size and locomotor habits should be considered [97]. Despite this general trend among primates, Jungers’ results [97] suggest that IMI is independent of body mass within callitrichids. This variable IMI scaling pattern in callitrichids may cause comparisons of IMI with other primate groups to be misleading. Additionally, as element proportions within each limb may vary, comparing a humerus to femur size ratio to the IMI would require extra caution.

Much like IMI, the humerus to femur size ratio is low for *Callimico goeldii* [78]. We also calculate a low size ratio within the *Callithrix* clade. A high IMI value in *Cebuella* was noted by Garbino [65], in agreement with the size ratio that we observed. It has been suggested that both high and low IMI might be associated with VCL within Callitrichidae, indicating a divergent adaptive path [39]. The elongated hindlimb observed in *Callimico* may provide a longer propulsive phase, allowing for greater speed at take off and thus increasing the potential for larger distances to be crossed while leaping, similar to the adaptation described for strepsirrhines specialized in VCL [39, 75, 98]. Alternatively, it is hypothesized that the elongated forelimb observed in *Leontocebus* allows for a longer deceleration phase, thus dampening the peak force during landing [39]. We have observed a relatively short humerus compared to the femur in the genus *Callithrix*, suggesting an adaptive response to VCL similar to the one observed in *Callimico*, and, to a lesser extent, also similar to what was deduced from quantifying IMI [65, 78]. Nevertheless, the analysis of trunk-to-trunk leaping kinematics [78] determined that *Callithrix jacchus* uses a leaping pattern similar to the one used by *Cebuella*: both present low angle and low velocity at take-off, thus losing more height during flight when compared to a jump of a similar distance by *Callimico* [78]. Thus *Callithrix jacchus* and *Cebuella* are expected to rely on multiple shorter jumps, instead of covering a long distance in a single trunk-to-trunk leap [33, 78, 98].

We found that size ratio in the whole *Leontocebus* genus is greater than in *Callimico* and *Callithrix*, and lower than in *Cebuella*. Garber [33] measured VCL habits (preferred jump distance) *in natura* that place *Leontocebus weddelli* in between *Callimico* and *Cebuella*.

The species of *Mico* included in this study have a similar ratio to *Leontocebus*. Moreover, the humeral shape tends to be similar suggesting an ecological similarity between *Mico* and *Leontocebus* (Fig. 4A). Correspondingly, the distribution area of the two genera is generally separate, as would happen if the two were competing for the same resources. It has been previously suggested that a competitive interaction might exist between the small-sized tamarin (*Leontocebus*) and *Mico* [38]. Nevertheless, overlaps in distribution exist in some areas [26, 99]. In these areas, they form mixed-species troops (*Mico emiliae* and *Leontocebus weddelli*, [100]; *Leontocebus weddelli*, *Mico melanurus*, and *Mico rondoni*; [101]). Thus the differences in distribution may be a result of evolutionary history rather than mutual exclusion.

### Evolution of shape in callitrichid proximal limb long bones

#### Global variation

The first two PCA axes for both the humerus and the femur are similarly structured: the *Leontopithecus* species are at an extreme, the HL tamarins occupy a central area, and all VCL species are spread along both axes to cover a large area. The case of *Callithrix kuhllii* must be considered with caution as it differs strongly from the other species of the genus *Callithrix*. This species is represented in our sample by a single specimen of unknown origin. The morphological separation of the genus *Leontopithecus* is well documented: greater body weight compared to other Callitrichidae [48], relative long hands [42] and derived incisors [24] that are linked with its derived food-seeking strategies and peculiar manipulative abilities [35, 47, 58, 59]. The slender humerus and femur, straight humeral shaft, and the small lesser trochanter of the femur are congruent with a reduced demand on shoulder flexors and hip adductors when compared to VCL species. The narrow and shallow trochlea reduces passive elbow stability in favor of mobility, requiring a more active control of stability [102, 103].

#### Humerus

The shape of the humeral head differs between the VCL and HL groups (Fig. 5A). The articular surface of the humeral head expands more in the disto-caudal direction in HL species, which may allow greater retraction of the humerus. In contrast, the more proximally oriented humeral head of VCL species (i.e., the orientation of the humeral head is more in line with the humeral shaft) places the greater tubercle in a relatively lower position, apparently allowing a greater degree of freedom of the humero-scapular articulation during shoulder extension. This has also been described in VCL strepsirrhine species [87, 104], although a study of joint kinematics during these two locomotor behaviors is currently lacking for Callitrichidae. The distal articular surfaces of the humerus also differ between the groups. The more expanded trochlea, particularly in its medial margin, observed in HL might increase elbow joint stability during flexion–extension while the smaller medial margin of the trochlea and the broader capitulum observed in VCL species may be assumed to result in greater pronation/supination abilities. Likewise, Szalay and Dagosto [87] highlighted the reduction in trochlea breadth observed in VCL strepsirrhines compared to other locomotor specialists. These authors also described the trochlea as expanding further towards the humeral shaft in VCL species. This is not the case in Callitrichidae, likely due to the difference in support substrate diameter preferences, and posture during clinging [24, 39]. Strepsirrhine species prefer small diameter vertical supports for clinging (although clawed strepsirrhines can cling onto large-sized trunks [75, 105]). Most Callitrichidae specialize in the use of large vertical supports (tree trunks), thanks to their claws. The larger the support, the greater the elbow angle [106]. Thus, it is unlikely that the vertical clinging locomotor behavior of callitrichids would induce the same need for an expanded trochlea in the antero-cranial direction as observed in Strepsirrhini. Again, the lack of knowledge of joint kinematics during locomotion in Callitrichidae limits conclusions about this characteristic.

#### Femur

The lesser trochanter is expanded in the VCL group (Fig. 5B). It provides the insertion for the iliopsoas, and this leg retractor plays a central role during clinging and has been shown to be enlarged in VCL specialists like strepsirrhine primates [74, 80]. Still, it may be suggested that an expanded lesser trochanter might provide a wider area of insertion for muscles that insert in direct proximity, such as the *vastus medialis* and *vastus intermedius* knee extensors that could be of adaptive value during leaping [107]. The actual predominance of one of these three muscles in shaping the lesser trochanter remains to be determined.

The femoral head is smaller in VCL species when compared to HL species. This reduction of the femoral head was also noted for *Cebuella* compared to other marmosets by Ford and Davis [81]. These authors suggest that it may be an adaptation to the constantly flexed hindlimb posture used during clinging.

The shape of the distal part of the medial condyle and the overall orientation of the tibiofemoral articulation during extension differ between the two groups. The orientation of the femur condyle observed in the VCL group induces a more everted position of the knee. Thus the knee articulation stays horizontal and the hip is less abducted, while the feet remain at a greater distance from each other. This is congruent with greater clinging abilities on large-diameter substrates like vertical trunks, but actual articular angles and associated forces remain to be investigated.

While VCL Strepsirrhine primates are often used to discuss the morphological changes observed in Callitrichidae, this article included, caution should be used when doing so. The fact that IMI in Callitrichidae does not follow the scaling pattern observed among primates, in addition to the fact that, unlike Strepsirrhini, Callitrichidae land hand first, may result in marked functional differences. In this context, Gebo [75] suggested that vertical clinging and leaping behavior should actually be seen as three distinct patterns: first that of tarsier and *Galago*, second the callitrichids and other keeled-nailed primates, and third the indriids and Lepilemur.

### Rate shifts in callitrichid limb evolution

#### Tamarins

The rates of evolution vary significantly across the phylogeny and especially among tamarins. The rates observed in the *Saguinus* clade are mostly low and are significantly lower than the tree average at the root of the *Saguinus* clade for humerus size, and for the size and shape of the femur. The clade that separates the *midas* group from the *oedipus* group is also significantly lower for both humeral and femoral size. These low evolutionary rates appear in a group that is almost exclusively composed of HL species. However, feeding diversity is dispersed throughout the phylogeny of the *Saguinus* clade with some species preferentially eating the reproductive parts of plants (> 70% of fruits and flowers; *S. oedipus* [72]; *S. niger* [71]; *S. labiatus* [34]; *S. leucopus* [70, 108]), and others consuming a greater amount of animal prey (> 30%; *S. bicolor* [109]; *S. midas* [47]; *S. geoffroyi* [36]; *S. mystax* [60]; *S. imperator* [110]). Thus, despite the relative shape homogeneity across the genus and the low evolutionary rates observed, the group exhibits considerable ecological diversity. The large tamarin morphotype (*Saguinus*) appears therefore to be a generalist morphology, allowing diversification through other means than locomotor niches, for example through food resources, cranial or dental morphology, or behavior. The role of this ecological segregation between closely related species in the diversification of the clade requires further investigation.

The *Leontocebus* clade is marked by increased evolutionary rates in all variables, and is significant for humeral size and shape, femoral shape, and the ratio between humerus and femur size (see Additional file 1: Table S4 and Fig. S4). The apparition of VCL behavior in this genus can thus be seen as a key innovation that resulted in an opportunity to exploit new resources. The genus *Leontocebus* shows differences in food sources between species. *Leontocebus weddelli* can be highly frugivore (up to 84% in the dry season [64]) whereas other species can be highly carnivore, such as *Leontocebus fuscicollis* (up to 48%; [60]). Moreover, the diet can vary between populations of the same species, or even within the same group, depending on the season [34, 60, 64, 111]. In opposition to what has been previously observed during specialization [112], we do not observe low evolutionary rates after the apparition of VCL abilities. The addition of a new food resource to the species repertoire (here vertical trunk insects and gum) might be the reason for the greater evolutionary rates observed in this group by broadening its ecological niche. The fact that this genus can form mixed groups with other Callitrichidae species tends to confirm this access to a new feeding niche [39, 44, 45, 99, 113].

#### Marmosets

We found high evolutionary rates for humeral and femoral shape changes within *Callithrix*, reaching significance only for the node separating *Callithrix kuhllii* from the *C. penicillata/C. jacchus* clade. The *C. penicillata/C. jacchus* clade is the group where one of the highest degrees of tree gouging specialization has been described and is considered to be an adaptation to the highly seasonal food resources available in the dry forests located in north-eastearn and central Brazil [27]. In this environment, fruit is not available year-round, thus the development of a high degree of gummivory was a key adaption [27]. The strong adaptive constraints induced by this new environment may have resulted in rapid morphological changes within the *C. penicillata/C. jacchus* clade. Still, as mentioned previously, only one specimen was obtained for *C. kuhllii* and it appears to diverge from the rest of the sample. Thus the high evolutionary rates at the node separating *Callithrix kuhllii* from the *C. penicillata/C. jacchus* clade may be due to an artifact driven by a single specimen.

## Conclusion

Quantified shape descriptors were used to investigate the shape evolution of the humerus and the femur associated with VCL behavior, which has appeared on two separate occasions within the evolution of the Callitrichidae.

Besides the phylogenetic inertia, VCL callitrichids share anatomical similarities that are functionally relevant. The distal articular surfaces of the humerus differ between HL and VCL species, similar to what was noted for other groups previously. The narrower trochlea may contribute to greater mobility in VCL species while the wider one visible in HL species appears to provide greater elbow stability. The femur differs in VCL species by exhibiting a relatively small femoral head, an expanded lesser trochanter, and the distal portion of the condyles are more laterally oriented. Functional interpretation suggests that these differences reflect the increased role of clinging in these species. Leaping probably involves the highest peak forces the musculoskeletal system encounters. However, these species also spend a lot of time clinging. Thus adaptations to clinging potentially yield large energetic savings and unsurprisingly seem to contribute to the evolution of limb long bone shape.

The lack of available comparative analyses of limb mechanics during trunk-to-trunk leaping in Callitrichidae limits our interpretation. Moreover, we are not aware of any published analyses quantifying bone strains during clinging behavior. The understanding of the evolution of the Callitrichidae would greatly benefit from such comparative experimental studies. More work of this kind has been conducted in Strepsirrhine primates but the marked differences in clinging and landing behaviors limit analogies between the two groups. Future work would greatly benefit from more quantitative data on locomotion to compliment research on locomotor group partition, which is arbitrary by definition. Only homogeneously quantified information would allow detailed morphological correlates to be identified. Moreover, information on the reasons for locomotion (moving toward a fruit feeding site, pursuing prey, escaping a predator) should be considered. The locomotor choices might differ during different circumstances. Moving between feeding sites might likely involve lower speed movements with a low falling risk to minimize the energetic cost, in comparison to escaping a predator, where an “all-out” strategy might be preferred.

This study has demonstrated significant differences in evolutionary rates within the Callitrichidae. The transition to VCL behavior within callitrichids (specifically the *Leontocebus* clade) appears to have been an opportunity for radiation, as opposed to a specialization that limits morphological diversity. In contrast, the genus *Saguinus* has primarily maintained a preference for HL and shows low evolutionary rates of change in terms of size and morphology of the humerus and femur, respectively, despite the diversity in feeding behavior. This suggests that this genus has reached a local fitness optimum of the locomotor apparatus.

## Methods

### Specimens

The 58 specimens analysed in this study were obtained from the Field Museum of Chicago, the American Museum of Natural History of New York, and the Museum d’Histoire Naturelle of Paris. To get a minimal estimate of intra-specific variability, up to three specimens per species were sampled, when available, from the collections. Microfocus computed tomography (µCT) scans were realized at the Prof. Luo Lab at the University of Chicago, at the Shared Materials Instrumentation Facility at Duke University, and the μCT facilities of the Montpellier Resources Imagerie at Université de Montpellier, respectively. The resolutions used vary between 12.6 and 18 µm depending on specimen size and scanning facility. Details on specimens, scanning resolution, scanning apparatus and operators are available in Additional file 2.

### Scan processing and landmark digitization

The μCT scans were segmented and surface models were extracted using Amira software (Thermo Fisher Scientific, version 6.0.0 [114]). The inner structure of the surface models was removed using Meshlab [115]. Final tidying and decimation of the surface models to 500,000 triangles were made under Geomagic wrap (3D Systems 2017).

Anatomical landmarks and curves were digitized using Morphodig 1.5.4 [116]. For a detailed map and designation of digitized anatomical landmarks and curves, please refer to Additional file 1: Tables S1–2 and Figs. S1–2. All specimens were measured twice to reduce potential digitization errors. One scan for each bone was arbitrarily chosen to serve as a template to allow semi-automated placement of surface sliding landmarks onto all surface models. The specimen *Callithrix argentata* AMNH 184689 was chosen as a template for the humerus and the specimen *Callimico goeldii* FMNH 153714 for the femur. The same points and curves, as in all specimens, were placed on template and surface sliding landmarks were digitized using IDAV Landmark [117].

### Morphometric procedure

All subsequent morphometric procedures and analyses were done using R [118]. Curves were resampled to the number detailed in Additional file 1: Table S3 using the ‘subsampl.inter’ function published in Botton-Divet et al. [16]. All functions used for patching, sliding, and superimposition are included in the ‘Morpho’ package for R [119]. Surface sliding landmarks were patched on all specimens using the ‘placePatch’ function, then specimens were relaxed against the template model under the minimum bending energy criterion using the ‘relaxLM’ function. Finally, the specimens were relaxed against the Procrustes consensus of the dataset for three iterations using the ‘slider3d’ function also minimizing bending energy. Slid specimens were then superimposed (generalized Procrustes analysis [120, 121]) using the ‘procSym’ function. To allow comparative analyses, a mean per species was computed by superimposing all specimens of the same species. The Procrustes consensus multiplied by the mean of centroid sizes was then used as the mean form of a species for subsequent analyses.

### Ecological categories and backbone phylogeny

Based on data available in the literature, we divided species into two categories, one for species with significant use of vertical clinging and leaping (VCL), one for preferential HL species based on the number of leaps to and from vertically oriented supports. Classification sources for each species can be found in Table 1. Considering the absence of quantitative data for the use of vertical support during locomotion for many species, and the differences in the protocols used for the available quantification, the classification of these species relies on qualitative information available in the literature. We are grateful to Prof. Eckhard Heymann (German Primate Center, Göttingen, Germany) for his help and comments on this classification attempt.

To use phylogenetic comparative methods, we built a composite phylogeny using the one published by Aristide et al. [73] as a backbone. We used Matauschek et al. [122] for the species within the *Leontocebus* genus and the time tree of life [123] was used as a source for *Mico melanurus*, *Saguinus leucopus,* and *Saguinus niger*. Branch lengths for added taxa were computed by scaling the branches from the above sources to match the backbone phylogeny [73]. In order to add a taxon, the branch lengths of the closest related species present in both the backbone and patch phylogenies were used to compute a scaling factor by cross multiplication. The added species may then be integrated into the backbone phylogeny with scaled branch lengths. *Leontocebus nigricollis* was also replaced by *Leontocebus fuscus*, as it is the closest relative species according to Cropp et al. [124] and Matauschek et al. [122].

Reconstruction of ancestral states is problematic given the repartition of the VCL versus HL character traits across the phylogeny (Fig. 1). An ancestral state reconstruction using parsimony criterion (function ‘asr_max_parsimony’ from the ‘castor’ R package [125]) leads to an unresolved state at the root of the phylogeny (a probability of 0.5 for both VCL and HL). The use of the likelihood method with stochastic character mapping using the ‘make.simmap’ function from the ‘phytools’ R package [126], with equal rate parameter and 1000 iterations results in posterior probabilities at the initial node of 0.45 and 0.55 for HL and VCL respectively. This result is highly influenced by the long branch that supports the *Leontopithecus* genus. Thus the result of such an analysis remains unreliable in this context. To overcome this we base the polarisation of the character on a functional argument. We hypothesize that clinging is a prerequisite to VCL. All Callitrichidae can cling to a certain extent, but trunk-to-trunk leaping is only adopted by some species [24]. VCL is therefore regarded as a derived state in the group.

### Statistics and assessment of evolutionary rate shifts

Differences in size between the two ecological categories were tested using two-sided t-test (‘t.test’ function in R) on log-transformed centroid size for both the humerus and femur. These differences were tested while taking phylogeny into account by using a phylogenetic ANOVA [127] using the ‘phylANOVA’ function from the ‘phytools’ package [126] with default settings.

In order to assess how strongly our data are driven by evolutionary history, and thus how strong the functional signal is, phylogenetic signal was measured. On centroid size (log), phylogenetic signal was quantified using Blomberg’s K statistic [128] and tested using the method from Ives et al. [129] with 10,000 iterations, using the ‘phylosig’ function implemented in the ‘phytools’ package [126]. Phylogenetic signals of shapes were measured using K_mult_ [130] and tested using the ‘physig’ function from the ‘geomorph’ package (version 3.3.1 [131]) with 10,000 iterations.

The effect of size (allometry), locomotor categories and the interaction of these on humeral and femoral shape was tested using MANCOVA. For this analysis, data dimensionality was reduced by selecting the first PCA axes gathering 95% of the total variance (14 and 15 axes for humerus and femur, respectively).

We computed the morphological difference between the two ecological groups by using between-group PCA. We evaluated the group separation by reclassification using the ‘typprobClass’ function from the ‘Morpho’ package [119] with cross-validation using Wilson’s [132] method for small samples. The significance of the between-group differences obtained from the between-group PCA was estimated by permutation test of the Euclidean distance between group means with 10,000 iterations. We visualized associated shape deformations by computing shape coordinates on the extreme of the axes and warping a dummy bone to the computed landmark values.

To test for changes in the evolutionary rates across the whole group we used the method developed by Castiglione et al. [94]. This method is based on Phylogenetic ridge regression [133] and measures the rate of evolutionary change for each branch, allowing the mean for each clade to be computed and then compared to the overall rate observed in the rest of the tree. The significance of rate difference between the considered clade and the rest of the tree is tested by randomization. We ran the ‘search.shift’ function [94] from the package ‘RRphylo’ [134] with the “clade” option on all clades smaller than one half of the tree.

## Supplementary Information

**Additional file 1: Table S1.** Designation of the anatomical landmarks used for the humerus. **Fig. S1.** Location of the anatomical landmarks used for the humerus. **Table S2.** Designation of the anatomical landmarks used for the femur. **Fig. S2.** Location of the anatomical landmarks used for the femur. **Table S3.** Number of curve sliding landmarks per curve for humerus and femur. **Fig. S3.** Deformations associated with PCA axes. **Table S4.** Test for evolutive rate shift in shape across the phylogeny. **Fig. S4.** Node numbering used in the Table S4.

**Additional file 2.** Details on specimens, scanning resolution, scanning apparatus and operators.

## Data Availability

All surface models are available through the Morphosource portal (https://www.morphosource.org). The identification numbers (ID) of the surface models are provided in Additional file 2.

## Abbreviations

HL: Horizontal leaping
VCL: Vertical clinging and leaping
PCA: Principal component analysis
PC: Principal component
µCT: Microfocus computed tomography
IMI: Intermembral Index
MANCOVA: Multivariate analysis of covariance
K_mult_: Multivariate extension of Blomberg’s Kappa as defined in [135]
3D: Three-dimensional
