# Supplementary material for: Vertical clinging and leaping induced evolutionary rate shifts in postcranial evolution of tamarins and marmosets (Primates, Callitrichidae)
Source: BMC Ecol Evol. 2021 Jun 25;21:132. doi: 10.1186/s12862-021-01848-z (PMC8235625; doi:10.1186/s12862-021-01848-z)
Supplement: Supplementary file 1 [file 12862_2021_1848_MOESM1_ESM.odt]

Supplementary Material 1

| Tab. S1 | Designation of the anatomical landmarks used for the humerus. |  |
| --- | --- | --- |
| Fig. S1 | Location of the anatomical landmarks used for the humerus. |  |
| Tab. S2 | Designation of the anatomical landmarks used for the femur. |  |
| Fig. S2 | Location of the anatomical landmarks used for the femur. |  |
| Tab. S3 | Number of curve sliding landmarks per curve for humerus and femur. |  |
| Fig. S3 | Deformations associated with PCA axes. |  |
| Tab. S4 | Test for evolutive rate shift in shape across the phylogeny. |  |
| Fig. S4 | Node numbering used in the Tab. S4 |  |

Table S1. Designation of the anatomical landmarks used for the humerus. Please refer to Fig. S1 for a visual representation.

| # | Designation |
| --- | --- |
| 1 | Most mediocranial point of the margin of the articular surface of the humeral head |
| 2 | Laterocaudal junction of the caudal lip of the femoral head with the tuberosita teres minor |
| 3 | Contact point between the greater trochanter crest and the insertion crest of the supraspinatus |
| 4 | Contact point between the the insertion crest of the supraspinatus and the one of the infraspinatus |
| 5 | Most proximomedial point of the cranial margin of the trochlea |
| 6 | Junction between the cranial margin of the trochlea and the crest between coronoid and radial fossa |
| 7 | Most proximolateral point of the cranial margin of the capitulum |
| 8 | Distolateral inflexion (maximum curvature) of the articular facet margin |
| 9 | Most proximolateral point of the caudal margin of the trochlea |
| 10 | Most proximomedial point of the caudal margin of the trochlea |
| 11 | Distomedial inflexion (maximum curvature) of the articular facet margin |
| 12 | Most craniodistal point of the medial supracondylar crest |
| 13 | Most caudodistal point of the medial supracondylar crest |
| 14 | Most proximomedial point of the medial supracondylar crest |
| 15 | Most laterodistal point of the lateral supracondylar crest |
| 16 | Lateral tip of the of the lateral supracondylar crest |
| 17 | Bottom of the olecranon fossa |
| 18 | Bottom of the radial fossa |

Figure S1. Location of the anatomical landmarks used for the humerus. Please refer to Tab. S2 for designation.

Table S2. Designation of the anatomical landmarks used for the femur. Please refer to Fig. S2 for a visual representation.

| # | Designation |
| --- | --- |
| 1 | Most proximal tip of the greater trochanter |
| 2 | Proximal insertion of the third trochanter |
| 3 | Distal insertion of the third trochanter |
| 4 | Most proximal point of the lateral facet of the the lesser trochanter |
| 5 | Most distal point of the lateral facet of the the lesser trochanter |
| 6 | Cranioproximal inflexion of the femoral head margin |
| 7 | Most cranial point of the distal facet of the greater trochanter |
| 8 | Most mediocranial point of the greater trochanter cranial ridge |
| 9 | Most craniolateral point of the greater trochanter cranial ridge |
| 10 | Most proximomedial point of the cranial margin of the patellar groove |
| 11 | Most proximolateral point of the cranial margin of the patellar groove |
| 12 | Point of contact between the lateral margin of the lateral condyle and the lateral margin of the patelar groove |
| 13 | Most proximolateral point of the caudal margin of the lateral condyle |
| 14 | Most proximomedial point of the caudal margin of the lateral condyle |
| 15 | Most cranial point of the distal margin of the trochlea between the condyles |
| 16 | Most proximolateral point of the caudal margin of the medial condyle |
| 17 | Most proximomedial point of the caudal margin of the medial condyle |
| 18 | Point of contact between the medial margin of the medial condyle and the lateral margin of the patelar groove |
| 19 | Most cranial point of the facet that articulate with the lateral sesamoid |
| 20 | Most cranial point of the facet that articulate with the medial sesamoid |
| 21 | Bottom of the depression of the medial colateral ligament insertion. |
| 22 | Bottom of the depression of the popliteus tendon insertion |

Figure S2. Location of the anatomical landmarks used for the femur. Please refer to Tab. S3 for designation.

Table S3. Number of curve sliding landmarks per curve for humerus and femur. Please refer to Fig. S1 (humerus) S2 (femur) for a visual representation and corresponding letters.

| Humerus | Femur |
| --- | --- |
| A | 20 | A | 20 |
| B | 6 | B | 5 |
| C | 20 | C | 6 |
| D | 3 | D | 5 |
| E | 10 | E | 10 |
| F | 5 | F | 6 |
| G | 7 | G | 10 |
| H | 7 | H | 12 |
| I | 7 | I | 6 |
| J | 5 | J | 8 |
| K | 5 | K | 8 |
| L | 7 | L | 6 |
| M | 5 | M | 12 |
| N | 20 | N | 10 |
| O | 4 |  |  |
| P | 5 |  |  |

Figure S3. Deformations associated with PCA axes. A: humerus first PC; B: humerus second PC; C: femur first PC, D: femur second PC. Blue shapes are for the negative side of the axis, red for the positive one. Views from left to right: caudal, medial, cranial, lateral.

The first axis of the PCA on humeral shape separate species with robust bones and proportionally large epiphyses on the positive side from species with relatively slenderer bone and smaller epiphyses. Species on the positive side tends to have shafts that seems curved in the parasagital plane as a result of a more developed and distal deltoid tuberosity. In the distal area the greater proportional size of the epiphyse observed in species with high PC values means a greater lateral supracondylar crest that expand more toward the proximal and lateral directions. In these species, the medial epicondyle is expended medially.

The second PC axis on humeral shape separate the species with bone shaft bent caudally around the deltoid tuberosity on the negative side from species with a straighter bone shaft on the positive one. Species with positive values present an humeral head tilted toward the medial side compared to species with low values. Species with higher values also tend to have a medial epicondyle less distally oriented.

The first PC axis of the femoral shape separate species with proportionally greater epiphyses on the negative side from species with relatively smaller ones on the positive side. Species on the negative side also show a medial distal condyle larger than the lateral one, resulting in a distal articular plane of the femur tilted toward the medial side (Fig.5 A). Additionally the species scoring on the negative sit of the first PC axis tend to present a femoral shaft with a double curvature in the parasagittal plane, when species on the positive side of the axis present a single curvature shaft.

The second PC axis of the femoral shape shows species with proportionally greater femoral head and smaller third trochanter on the positive side of the axis. Specimens on the positive side of the axis also present a proportionally greater medial distal condyle.

Table S4. Test for evolutive rate shift in shape across the phylogeny. Significant p-value are >0,975 for greater rates and <0,025 for smaller rates. Please refer to Fig. S4 for node numbering

|  | Humerus |  |  |  | Femur |  |  |  | Size ratio |  |
| --- | --- | --- | --- | --- | --- | --- | --- | --- | --- | --- |
|  | shape |  | size |  | shape |  | size |  |  |  |
| Node | Rate diff. | p.val | Rate diff. | p.val | Rate diff. | p.val | Rate diff. | p.val | Rate diff. | p.val |
| 27 | -0.00310571 | 0.1915 | 0.00610890 | 0.9597 | 0.00086968 | 0.6562 | 0.00865522 | 0.9956 | -0.00084693 | 0.1205 |
| 28 | 0.00247527 | 0.8473 | 0.02271524 | 0.9312 | 0.00836766 | 0.8985 | 0.02161406 | 0.9427 | -0.00112737 | 0.2113 |
| 29 | -0.00246867 | 0.2919 | 0.00333807 | 0.8083 | 0.00043679 | 0.5844 | 0.00706815 | 0.9716 | -0.00057742 | 0.2725 |
| 30 | -0.00131561 | 0.4196 | 0.00416854 | 0.8427 | 0.00149375 | 0.7265 | 0.00754664 | 0.9693 | -0.00045347 | 0.3818 |
| 31 | -0.00575779 | 0.0913 | 0.00793068 | 0.8810 | -0.00319658 | 0.2280 | 0.01465721 | 0.9820 | -0.00071931 | 0.3924 |
| 32 | -0.00503299 | 0.2345 | -0.00523573 | 0.3166 | -0.00259122 | 0.3988 | -0.00018979 | 0.5674 | -0.00079841 | 0.3552 |
| 33 | -0.00319564 | 0.5372 | 0.00088283 | 0.6797 | -0.00110764 | 0.6621 | 0.00727495 | 0.8049 | -0.00059782 | 0.5579 |
| 34 | 0.00577828 | 0.7678 | 0.00382692 | 0.7351 | 0.00793386 | 0.9580 | 0.00348837 | 0.7357 | 0.00011069 | 0.7587 |
| 35 | 0.00979494 | 0.8425 | -0.00161300 | 0.5201 | 0.01233902 | 0.9778 | 0.00273583 | 0.6819 | 0.00011901 | 0.8272 |
| 36 | 0.00662259 | 0.8929 | -0.00430043 | 0.4880 | 0.01015850 | 0.9120 | 0.01051135 | 0.8454 | 0.00066748 | 0.9171 |
| 38 | 0.01822373 | 0.9966 | 0.01078758 | 0.9635 | 0.01049155 | 0.9940 | 0.00324708 | 0.7441 | 0.00404923 | 0.9983 |
| 39 | 0.02528520 | 0.9987 | 0.01611103 | 0.9842 | 0.01458213 | 0.9975 | 0.00803401 | 0.8928 | 0.00546425 | 0.9992 |
| 40 | 0.03897087 | 0.9997 | 0.02705482 | 0.9965 | 0.02286979 | 0.9994 | 0.01681109 | 0.9690 | 0.00840849 | 0.9998 |
| 41 | 0.07749658 | 1.0000 | 0.05686002 | 1.0000 | 0.04502203 | 1.0000 | 0.04003517 | 0.9959 | 0.01714801 | 1.0000 |
| 42 | -0.00460508 | 0.1137 | -0.00866060 | 0.0056 | -0.00492278 | 0.0049 | -0.00735356 | 0.0134 | -0.00104180 | 0.0989 |
| 43 | -0.00597290 | 0.1277 | -0.00207494 | 0.4995 | -0.00427339 | 0.1325 | 0.00280272 | 0.6856 | -0.00066091 | 0.4843 |
| 44 | -0.00436347 | 0.4461 | 0.00390170 | 0.7365 | -0.00282876 | 0.5046 | 0.01471814 | 0.8883 | -0.00000156 | 0.8473 |
| 45 | -0.00175491 | 0.4829 | -0.00813653 | 0.0248 | -0.00332162 | 0.1226 | -0.00862287 | 0.0132 | -0.00079456 | 0.2758 |
| 46 | 0.00080279 | 0.7656 | -0.00689385 | 0.2028 | -0.00277876 | 0.3786 | -0.01102941 | 0.0133 | -0.00029028 | 0.7369 |
| 47 | 0.00742904 | 0.9022 | 0.00028183 | 0.6625 | -0.00098075 | 0.6665 | -0.01150412 | 0.0447 | 0.00028029 | 0.8936 |
| 48 | -0.00111927 | 0.6623 | -0.00603231 | 0.2630 | -0.00168923 | 0.4964 | -0.00318197 | 0.3874 | -0.00076312 | 0.3895 |
| 49 | 0.00067411 | 0.7918 | -0.00261017 | 0.5677 | -0.00158216 | 0.6363 | -0.00476347 | 0.4643 | -0.00111674 | 0.2199 |

Figure S4. Node numbering used in the Tab. S3 of the present supplementary materials.
